# Prevalence of overlap syndromes and symptoms in pediatric functional dyspepsia

**DOI:** 10.1186/s12876-016-0495-3

**Published:** 2016-07-25

**Authors:** Craig A. Friesen, John M. Rosen, Jennifer V. Schurman

**Affiliations:** 1 Division of Gastroenterology, Hepatology, and Nutrition, Children’s Mercy Kansas City, 2401 Gillham Road, Kansas City, MO 64108 USA; 2 Division of Developmental and Behavioral Sciences, Children’s Mercy Kansas City, 2401 Gillham Road, Kansas City, MO 64108 USA

**Keywords:** Functional dyspepsia, Irritable bowel syndrome, Gastroesophageal reflux, Overactive bladder syndrome

## Abstract

**Background:**

The purpose was to evaluate the overlap frequency of irritable bowel syndrome (IBS), gastroesophageal reflux disease (GERD), and overactive bladder syndrome (OBS), as well as other gastrointestinal and systemic symptoms, in functional dyspepsia (FD). Additionally, we sought to determine whether adult Rome III FD subtypes were uniquely related to overlap syndromes or symptoms.

**Methods:**

The study was a retrospective review of 100 consecutive pediatric patients, age 8–17 years, diagnosed with FD. All had completed a standardized medical history including gastrointestinal and systemic symptoms as well as specific symptoms related to GERD and OBS. The frequency of overlap with IBS, GERD, and OBS were determined for the whole group and for those fulfilling adult FD subtype criteria. Individual symptoms were also compared by FD subtype.

**Results:**

Overlap IBS was present in 33 % of the FD patients. At least one GERD symptom was present in 74 % of patients with 41 % reporting heartburn. At least one OBS symptom was present in 44 % of patients with 29 % reporting urinary urgency. Other than pain, the most common reported gastrointestinal symptom was nausea (86 %). Systemic symptoms were common. Overlap syndromes/symptoms did not vary by FD subtype. Postprandial distress syndrome was associated with pain with eating, weight loss, and waking at night to have a stool.

**Conclusions:**

FD is a heterogeneous condition in children and adolescents with significant variability in the presence of gastrointestinal and non-gastrointestinal symptoms and overlap syndromes. Varying symptom profiles need to be accounted for and analyzed in studies involving subjects with FD.

## Background

There are a number of pain-associated functional gastrointestinal disorders (FGIDs) defined by Rome III for children/adolescents, namely irritable bowel syndrome (IBS), functional dyspepsia (FD), abdominal migraines, functional abdominal pain, and functional abdominal pain syndrome [1]. In adults, there are two recognized FD subtypes, postprandial distress syndrome (PDS; defined by the presence of early satiety preventing completing a normal size meal or fullness following a normal sized meal) and epigastric pain syndrome (EPS; defined by the presence of pain or burning localized to the epigastrium) [2] These subtypes are not recognized in the pediatric Rome III criteria, although there is some evidence of their existence and indication that the distinction may be clinically meaningful in youth as well [3, 4]. For example, PDS-related symptoms (i.e., early satiety and/or bloating following a meal) have been associated with increased mucosal mast cells, anxiety, and depression in pediatric FD [4].

Most children and adolescents with chronic abdominal pain fulfill criteria for an FGID with FD and IBS being the two most common [1, 5, 6]. These diagnoses form the entry criteria for most abdominal pain studies and therapeutic trials. Utilization of these criteria for research (or clinical practice) presents challenges given some inherent ambiguity in the criteria, inconsistent application of the criteria, and differences in symptom reports between the patients and their parents or guardians [7]. Additionally, there may be overlap between FGIDs or overlap between FGIDs and other symptoms or conditions which may influence study results or therapeutic response. For adults, FD is well known to overlap with IBS, as well as other conditions such as gastroesophageal reflux disease (GERD) and overactive bladder syndrome (OBS) [8–11]. Overlap syndromes may be associated with greater psychopathology and electromechanical dysfunction [12, 13]. We have previously found that FD/IBS overlap accounts for 30 % of youth presenting for initial evaluation of chronic abdominal pain [6]. This represents another area of variability in utilization of Rome criteria as some investigators diagnose overlap FD/IBS while others presumably default to IBS when symptoms of both are present [7]. Overlap with GERD and OBS has been largely unstudied in a pediatric population. It is important to determine to what degree, if any, individual FGIDs overlap with other syndromes or symptoms in order to understand variability within a diagnostic category which may affect study results or patient outcomes.

The purpose of the current study was to evaluate the frequency of overlap IBS, potential GERD-related symptoms, and OBS-related symptoms, as well as other gastrointestinal and systemic symptoms, in a clinical sample of youth with FD. Additionally, we sought to determine the frequency of PDS and EPS in youth with FD, and whether these subtypes were uniquely related to overlap syndromes or other symptoms.

## Methods

### Study design

The current study was a retrospective chart review of pediatric patients who were identified as being diagnosed with FD after being seen for initial evaluation of chronic abdominal pain in a subspecialty abdominal pain clinic housed within a large Midwestern children’s hospital between September, 2014 and June, 2015. All data described below were collected as part of standard care and were abstracted from the medical record and entered into a research database for analysis in the current study. The study was approved by the institutional review board at Children’s Mercy Kansas City.

### Study cohort and exclusion criteria

All patients selected for inclusion had been evaluated in a single subspecialty abdominal pain clinic. Patients were 8–17 years, inclusive, and reported pain at least weekly for a minimum of 8 weeks per standard entry criteria for the clinic. The diagnosis of FD was determined according to Rome III criteria by a single board-certified pediatric gastroenterologist based on an extensive history and physical examination conducted with each child and family during the initial clinic visit [1]. All patients had undergone upper endoscopy with biopsy after failing to respond to acid reduction therapy of at least 2 weeks duration. All were without gross pathology (including nodularity, erosions, and ulceration. Per standard protocol at the participating hospital, all patients had at least 2 biopsies obtained from each of the lower one-third of the esophagus and the gastric antrum and at least four biopsies from the duodenum for histologic evaluation. All patients were negative for *Helicobacter pylori* on antral biopsy. Mild histologic esophagitis (peak eosinophils < 5/hpf) and/or mild chronic gastritis did not exclude the patient from selection. Patients diagnosed with other pathology, including eosinophilic gastroenteritis, celiac disease, inflammatory bowel disease, or giardiasis, were excluded from the study cohort. Eosinophilic gastritis and duodenitis were diagnosed at the discretion of the evaluating board-certified pathologist as no widely accepted criteria exist regarding eosinophil density.

### Data sources

#### Pathology

As a prerequisite for inclusion in the study cohort, patients had to have previous biopsies to evaluate for the presence of histologic esophagitis as assessed by a board certified pediatric pathologist as part of routine care. The pathology report provided the histologic data for analysis.

#### Gastrointestinal and systemic symptoms

All patients seen for initial evaluation in the abdominal pain clinic from which the sample was selected had completed a standardized medical history. This medical history included questions regarding the presence of upper abdominal pain or discomfort, as well as any relation to a change in stool frequency or consistency or a history of relief from stooling. In addition, specific locations of the pain were obtained by asking the patient to mark pain locations on a diagram of the abdomen. The nature of the pain (e.g. burning, crampy) was asked of each patient. Patients were specifically asked whether pain wakes them at night and whether pain increases after a meal. Patients also were asked about the presence of nausea and/or vomiting and whether nausea increases after a meal. Relevant to the issue of FD subtyping, patients were asked specifically about the presence of early satiety and postprandial bloating [2].

Patients also are asked about the presence of a number of other gastrointestinal and systemic symptoms. Gastrointestinal symptoms that are assessed include the presence of stool symptoms such as loose stools, hard stools, mucus in stools, blood in stools, and waking at night to have a stool, as well as the presence of excessive gas. Systemic symptoms that are assessed include hayfever, fatigue, headache, fainting, dizziness, chest pain, muscle pain, joint pain, mouth sores, weight loss, and fever. In addition to the above, patients are asked about the presence of 4 symptoms related to gastroesophageal reflux (bitter, salty, or sour taste in mouth; excessive belching or burping; regurgitation; heartburn) and 3 symptoms related to overactive bladder syndrome (increased frequency of urination; waking during the night to urinate; sudden urge to urinate).

Information from the medical history, once abstracted and entered into the research database, was used to evaluate dyspeptic symptoms and assign an FD subtype based on adult Rome III criteria. Patients were defined as having postprandial distress syndrome if they endorsed early satiety or postprandial bloating [2]. Patients were defined as having epigastric pain syndrome if they endorsed burning or pain localized only to the epigastric region [2]. Diagnoses of potentially overlapping symptoms and conditions (i.e., IBS, GERD, OBS) also were assigned based on symptom information abstracted from the patient’s medical history.

### Statistical analysis

Statistical analysis was performed using SPSS Version 20 (SPSS Inc, Chicago, IL). Frequencies were determined for individual symptoms and diagnoses. The frequencies of individual reflux symptoms were compared between patients with and without mild histologic esophagitis by chi-square analysis or Fisher’s exact tests, as appropriate given cell frequencies. The frequencies of an IBS diagnosis, reflux symptoms, and overactive bladder symptoms, respectively, were compared between genders, between patients <13 years of age and those ≥13 years of age, and between patients with and without PDS by chi-square analysis or Fisher’s exact tests, as appropriate given cell frequencies. A *p* value of <0.05 was considered statistically significant for all of the analyses. Given the exploratory nature of the current study, trends were reported for *p* values >0.05 but <0.07.

## Results

### Demographics of the study cohort

One hundred patients ranging in age from 8 to 17 years with a mean age of 13 years 9 months were evaluated. Seventy-six percent were female. Pain was reported to occur daily in 76 %, several times/week in 19 %, and weekly in 5 % of patients. Pain was described as intermittent by 60 % and continuous by 40 % of patients. For patients reporting nausea (*n* = 86), the nausea occurred daily in 36 % and at least weekly in 76 %. Sixty-three percent reported that nausea increased with eating. Frequencies of other selected gastrointestinal symptoms and systemic symptoms are shown in Table 1. All patients had failed to respond clinically to acid reduction therapy. Forty percent were treated with ranitidine (>6 mg/Kg/day up to 150 mg twice daily) and 72 % were treated with a proton pump inhibitor (PPI; >1 mg/Kg/day up to 40 mg twice daily for omeprazole, pantoprazole, and esomeprazole and up to 30 mg twice daily for lansoprazole). Twelve percent received courses of both ranitidine and a PPI. Of those treated with a PPI, 32 % received twice daily dosing and 21 % received multiple courses of different PPIs. As a requirement, all patients underwent upper endoscopy with biopsies. No patients had a previous upper gastrointestinal barium study, gastric emptying study, or pH-impedance study. No patients reported previous food allergy testing and while some patients were avoiding specific foods, none had been on a full elimination diet.

**Table 1:** Frequency of specific gastrointestinal and systemic symptoms in FD patients

Symptom	% Reporting Symptom
Waking at night with pain	64
Pain increased with eating	68
Increased appetite	4
Decreased appetite	61
Nausea	86
Vomiting	32
Early satiety	74
Postprandial bloating	62
Fatigue	47
Headache	66
Dizziness	53
Weight loss	30
Muscle pain	28
Joint pain	26

### FD subtypes

Seventy-two percent of patients met criteria for PDS. Bloating was reported by 62 % and early satiety by 74 % of all FD patients. Of those endorsing early satiety, 85 % reported that it prevented finishing a regular meal. While 52 % of all patients reported epigastric pain or burning, only 3 % localized this pain and/or burning only to the epigastrium as required to fulfill criteria for EPS. Twenty-five percent of patients met criteria for FD but not PDS or EPS subtypes.

### Overlap syndromes/symptoms in FD

One-third (33 %) of FD patients also fulfilled criteria for IBS. Frequencies for GERD-related symptoms are shown in Table 2. At least one symptom was reported by 74 %, 2 or more symptoms by 49 %, 3 or more symptoms by 29 %, and all 4 symptoms by 6 % of patients. Patients with mild histologic esophagitis reported higher frequencies compared to those with normal histology for bitter/salty taste (52 vs. 29 %; *p* = .046), belching/burping (62 vs. 22 %; *p* = .001), regurgitation (64 vs. 54 %; *p* = .042), and heartburn (71 vs. 32 %; *p* = .001). Frequencies for OBS-related symptoms are shown in Table 2. Of the patients reporting night time urination, 67 % reported that this occurred at least weekly. At least one symptom was reported by 44 %, 2 or more symptoms by 23 %, and all 3 symptoms by 8 %. No differences were found in the frequencies of overlap relative to age or gender with one exception. Specifically, IBS overlap was more common in females 13 years of age and older as compared to younger females (36 vs. 13 %; *p* = .037).

**Table 2:** Frequency of overlap syndromes and symptoms in FD patients

Syndrome/Symptom	% of patients
IBS	33
GERD: bitter, salty, or sour taste	35
GERD: excessive belching or burping	30
GERD: regurgitation	57
GERD: heartburn	41
OBS: increased frequency of urination	13
OBS: waking at night to urinate	33
OBS: sudden urge to urinate	29

### Overlap syndromes/symptoms by FD subtype

Patients fulfilling criteria for PDS (as compared to those who did not) were more likely to report pain with eating (79 vs. 43 %; *p* = .001), weight loss (36 vs. 14 %; *p* = .026), and waking at night to have a stool (27 vs. 7 %; *p* = .025). There also were trends for patients with PDS to be more likely to report nausea with eating (70 vs. 50 %; *p* = .052), pain relief with a stool (39 vs. 21 %; *p* = .069), mucus in the stool (17 vs. 4 %; *p* = .067), and increased gas (44 vs. 25 %; *p* = .066). Although other gastrointestinal and systemic symptoms were found to be relatively common in the broader group of patients with FD, no other correlations emerged between these symptoms and the PDS subtype. PDS also did not have any significant associations with any GERD-related or OBS-related symptoms. Associations with overlap syndromes/symptoms were not examined for EPS given the low base rate (3 %) in the study cohort.

## Discussion

The current study indicates that FD is a heterogeneous condition in children and adolescents. There is significant variability in the presence of gastrointestinal, as well as non-gastrointestinal, symptoms. It is important to understand this variability within diagnostic categories, and also within individual patients as each patient appears to have a somewhat unique symptom profile within the broad category of FD.

Overlap IBS was present in 33 % of the FD patients in the current study. Previously we found IBS overlap in 54 % of patients fulfilling FD criteria [6]. Consistent with these pediatric findings, IBS overlap has been reported in 33–56 % of adults with FD [14–16]. In adults, IBS occurs more often in those with FD (37 %) as compared to those without FD (7 %) [12]. IBS has been found to be more common with the PDS subtype in some, but not all, studies in adults [12, 15]. We found no association between overlapping IBS and PDS; however, the PDS subtype patients were more likely to report the individual symptom of waking at night to have a stool. There was also a trend towards increased reporting of pain improvement with a stool in the PDS subgroup. Associations with EPS could not be assessed given the low EPS frequency. In adults, FD/IBS overlap patients were more likely to be female. (13) We did not find any main effect of gender in our pediatric cohort, however, females 13 years of age and older were more likely to report overlap than were younger females (12 years of age and under).

Evaluating for the presence of GERD overlap in youth with FD presents a challenge, as there is no gold standard test for GERD. Symptoms and available tests lack high sensitivity or specificity. However, the North American Society for Pediatric Gastroenterology, Hepatology, and Nutrition guidelines concluded, based on expert opinion, that the diagnosis of GERD can be made in adolescents with typical heartburn symptoms [17]. Heartburn is considered to be a reasonably sensitive indicator of GERD in adults [18]. Heartburn was reported by 41 % of the patients in the current study. Although a definitive statement isn’t possible, our data suggest that reflux-like symptoms are common in youth with FD. The correlation between reflux-like symptoms and mild histologic esophagitis adds some support for these symptoms being related to GERD in at least some patients. Reported prevalence of GERD in adults with FD has ranged from 13.3 to 56 %, with the prevalence being near 50 % in most studies [12]. This overlap appears to be greater in those patients with non-erosive GERD (NERD) and, within this group, overlap is greatest among those with heartburn [8, 9]. It has been suggested that heartburn should be considered an integral part of the dyspepsia complex in adults [19].

Overactive bladder symptoms were common in FD patients in the current study, with at least one symptom present in 44 %. In adults, OAB is defined as a symptom complex of urinary urgency usually with urinary frequency and nocturia [10]. The hallmark of OAB in children is urgency. (20) In the current study, urgency was reported by 29 % of the patients. This is similar to the 20.5 % prevalence of OAB in adults with FD [11]. Pathophysiologic processes implicated in OAB are similar to those that appear relevant to FD (and IBS) including nerve hypersensitivity and mucosal mast cells [20–22].

The FD subtypes, PDS and EPS, are well recognized in adults but were not included in the pediatric FD criteria [1]. In adults, some studies have shown good separation of PDS and EPS while others have reported significant overlap of the two subtypes [23]. It appears that separation is better in the general population than in patients who seek medical care [24]. In one pediatric study, Turco and colleagues evaluated 100 pediatric patients with FD and found EPS alone in 17 %, PDS alone in 47 %, and overlap in 36 %; however, many of these patients crossed over their diagnosis between PDS and EPS over time [3]. In the current study, 72 % of our pediatric cohort met criteria for PDS, while only 3 % met criteria for EPS. Similar to our previous findings regarding EPS, 52 % of the patients in the current study reported epigastric pain or burning, but only 3 % localized this pain exclusively to the epigastrium, a requirement for diagnosing EPS under the Rome III adult criteria [4].

Adult data suggests that meal-related symptoms, including pain and nausea, may be the norm in FD [25]. The PDS subtype did have some associations with other GI symptoms in our pediatric cohort, including increased pain with eating (79 %) and weight loss (36 %), as well as a trend toward increased nausea with eating (70 %). Thus, the consequences of eating may be more widespread than just early satiety. In adult studies, nausea has been reported by 39–65 % of FD patients [24]. Overall, nausea was reported by 86 % of pediatric patients in the current study with 70 % reporting an increase in nausea with eating. In another pediatric abdominal pain study, nausea was reported by 87 % of subjects who met adult criteria for FD [26]. Thus, nausea appears to be a common component of FD in both adults and youth. However, nausea may be more associated with PDS than EPS in children [3]. Nausea is important to consider as it is associated with poor school functioning and greater social disability [26]. These meal related symptoms may also be associated with nutritional consequences. As noted above, weight loss was common in the current study being reported by 36 % of PDS patients. This is similar to adults where weight loss correlates most strongly with early satiety [27].

The main strength of the current study is the relatively large population size with symptom reporting performed in a standardized fashion across a wide array of gastrointestinal and non-gastrointestinal symptoms. The main limitation is that the study design did not allow evaluation of the natural history of these symptoms. It is known that symptoms may evolve across time and patients may change FD subtype classification [3].

## Conclusions

In sum, pediatric FD is a heterogeneous syndrome exhibiting variable symptoms from patient to patient with many, but not all, fulfilling criteria for PDS. The clinical presentation of FD also varies with regard to non-FD symptoms, exhibiting significant overlap with IBS as well as symptoms consistent with GERD and OBS. These varying symptom profiles need to be considered in providing care to FD patients. For example, an FD patient with overlap may have a different pathogenic process or respond differently to a treatment regimen than a patient with FD alone or FD with overlap GERD. Likewise, varying symptom profiles need to be accounted for and analyzed in studies involving subjects with FD. Clinical trials need to be designed to fit the patients that are actually encountered, rather than the clean diagnostic boxes we create.

## Abbreviations

EPS, epigastric pain syndrome; FD, functional dyspepsia; FGID, functional gastrointestinal disorder; GERD, gastroesophageal reflux disease; hpf, high power field; IBS, irritable bowel syndrome; OBS, overactive bladder syndrome; PDS, postprandial distress syndrome

## References

[1] Rasquin A, Di Lorenzo C, Forbes D, Guiraldes E, Hyams JS, Staiano A, Walker LS (2006). Childhood functional gastrointestinal disorders: child/adolescent. Gastroenterology.

[2] Tack J, Talley NJ, Camilleri M, Holtmann G, Hu P, Malagelada JR, Stanghellini V (2006). Functional gastroduodenal disorders. Gastroenterology.

[3] Turco R, Russo M, Martinelli M, Castiello R, Coppola V, Miele E, Staiano A. Do distinct functional dyspepsia subtypes exist in children? J Pediatr Gastroenterol Nutr 2015; doi: 10.1098/MPG.0000000000000944. 10.1097/MPG.0000000000000944 26284541

[4] Schurman JV, Singh M, Singh V, Neilan N, Friesen CA (2010). Symptoms and subtypes in pediatric functional dyspepsia: relation to mucosal inflammation and psychological functioning. J Pediatr Gastroenterol Nutr.

[5] Walker L, Lipani TA, Greene JW, Caines K, Stutts J, Polk DB, Caplan A, Rasquin-Weber A (2004). Recurrent abdominal pain: symptom subtypes based on the Rome II criteria for pediatric functional gastrointestinal disorders. J Pediatr Gastroenterol Nutr.

[6] Schurman JV, Friesen CA, Danda CE, Andre L, Welchert E, Lavenbarg T, Cocjin JT, Hyman PE (2005). Diagnosing functional abdominal pain with Rome III criteria: parent, child, and clinician agreement. J Pediatr Gastroenterol Nutr.

[7] Friesen CA, Schurman JV, Abdel-Rahman SM (2015). Present state and future challenges in pediatric abdominal pain therapeutics research: Looking beyond the forest. World J Gastrointest Pharmacol Ther.

[8] Quigley EM, Lacy BE (2013). Overlap of functional dyspepsia and GERD- diagnostic and treatment implications. Nat Rev Gastroenterol Hepatol.

[9] Yarandi SS, Christie J (2013). Functional dyspepsia in review: Pathophysiology and challenges in the diagnosis and management due to coexisting gastroesophageal reflux disease and irritable bowel syndrome. Gastroenterol Res Pract.

[10] Homma Y, Yoshida M, Seki N, Yokoyama O, Kakizaki H, Gotoh M, Yamanishi T, Yamaguchi O, Takeda M, Nishizawa O (2006). Symptom assessment tool for overactive bladder syndrome- overactive bladder symptom score. Urology.

[11] Matsuzaki J, Suzuki H, Fukushima Y, Hirata K, Fukuhara S, Okada S, Hibi T (2012). High frequency of overlap between functional dyspepsia and overactive bladder. Neurogastroenterol Motil.

[12] Fujiwara Y, Arakawa T (2014). Overlap in patients with dyspepsia/functional dyspepsia. J Neurogastroenterol Motil.

[13] Corsetti M, Caenepeel P, Fischler B, Janssens J, Tack J (2004). Impact of coexisting irritable bowel syndrome on symptoms and pathophysiological mechanisms in functional dyspepsia. Am J Gastroenterol.

[14] Wang AJ, Liao XH, Xiong LS, Peng S, Xiao YL, Liu SC, Hu PJ, Chen MH (2008). The clinical overlap between functional dyspepsia and irritable bowel syndrome based on Rome III criteria. BMC Gastroenterol.

[15] Xiong LS, Shi Q, Gong XR, Cui Y, Chen MH (2014). The spectra, symptom profiles and overlap of Rome III functional gastrointestinal disorders in a tertiary center in South China. J Dig Dis.

[16] Van Oudenhove L, Vandenberghe J, Vos R, Holvoet L, Tack J (2011). Factors associated with co-morbid irritable bowel syndrome and chronic fatigue-like symptoms in functional dyspepsia. Neurogastroenterol Motil.

[17] Vandenplas Y, Rudolph CD, Di Lorenzo C, Hassall E, Liptak G, Mazur L, Sondheimer J, Staiano A, Thomson M, Veereman-Wauters G, Wenzl TG (2009). Pediatric gastroesophageal reflux clinical practice guidelines: Joint recommendations of the North American Society for Pediatric Gastroenterology, Hepatology, and Nutrition (NASPGHAN) and the European Society for Pediatric Gastroenterology, Hepatology, and Nutrition (ESPGHAN). J Pediatr Gastroenterol Nutr.

[18] Lacy BE, Weiser K, Chertoff J, Fass R, Pandolfino JE, Richter JE, Rothstein RI, Spangler C, Vaezi MF (2010). The diagnosis of gastroesophageal reflux disease. Am J Med.

[19] Talley NJ (2013). Functional (non-ulcer) dyspepsia and gastroesophageal reflux disease: one not two diseases?. Am J Gastroenterol.

[20] Franco I (2012). Functional bladder problems in children: pathophysiology, diagnosis, and treatment. Pediatr Clin North Am.

[21] Gamper M, Regauer S, Welter J, Eberhard J, Viereck V (2015). Are mast cells still good biomarkers for bladder pain syndrome/interstitial cystitis?. J Urol.

[22] Liu HT, Shie JH, Chen SH, Wang YS, Kuo HC (2012). Differences in mast cell infiltration, E-cahedrin, and zonula occludens-1 expression between patients with overactive bladder and interstitial cystitis/bladder pain syndrome. Urology.

[23] Shin CM (2013). Overlap between postprandial distress and epigastric pain syndromes in functional dyspepsia: its implications for research and clinical practice. Am J Gastroenterol.

[24] Tack J, Talley NJ (2013). Functional dyspepsia- symptoms, definitions and validity of Rome III criteria. Nat Rev Gastroenterol Hepatol.

[25] Talley NJ (2015). Functional dyspepsia and the Rome criteria: a success story. Neurogastroenterol Motil.

[26] Kovacic K, Williams S, Li BU, Chelimsky G, Miranda A (2013). High prevalence of nausea in children with pain-associated functional gastrointestinal disorders: are Rome criteria applicable?. J Pediatr Gastroenterol Nutr.

[27] Tack J, Jones MP, Karamanolis G, Coulie B, Dubois D (2010). Symptom pattern and pathophysiologic correlates of weight loss in tertiary-referred functional dyspepsia. Neurogastroenterol Motil.

